# Platelet characteristics in extremely preterm infants after fatty acid supplementation: a randomized controlled trial

**DOI:** 10.1038/s41390-024-03775-3

**Published:** 2024-12-19

**Authors:** Pia Lundgren, Aldina Pivodic, Anders K. Nilsson, Gunnel Hellgren, Hanna Danielsson, Dirk Wackernagel, Ingrid Hansen Pupp, David Ley, Karin Sävman, Mattias Uhlén, Lois E. H. Smith, Ann Hellström

**Affiliations:** 1 The Sahlgrenska Centre for Pediatric Ophthalmology Research, Department of Clinical Neuroscience, Institute of Neuroscience and Physiology, Sahlgrenska Academy, University of Gothenburg, Gothenburg, Sweden; 2 Department of Ophthalmology, Sahlgrenska University Hospital, Region Västra Götaland, Gothenburg, Sweden; 3 Institute of Biomedicine, Sahlgrenska Academy, University of Gothenburg, Gothenburg, Sweden; 4 Centre for Translational Microbiome Research, Department of Microbiology, Tumor and Cell Biology, Karolinska Institutet, Stockholm, Sweden; 5 Sach’s Children’s and Youth Hospital, Södersjukhuset, Stockholm, Sweden; 6 Department of Clinical Science, Intervention and Technology (CLINTEC), Karolinska Institutet, Stockholm, Sweden; 7 Division of Neonatology, Department of Pediatrics, University Medical Center of the Johannes Gutenberg-University Mainz, Mainz, Germany; 8 Department of Clinical Sciences, Lund, Pediatrics, Lund University, and Skåne University Hospital, Lund, Sweden; 9 Department of Pediatrics, Institute of Clinical Sciences, Sahlgrenska Academy, University of Gothenburg, Gothenburg, Sweden; 10 Region Västra Götaland, Department of Neonatology, The Queen Silvia Children’s Hospital, Sahlgrenska University Hospital, Gothenburg, Sweden; 11 Science for Life Laboratory, Department of Protein Science, KTH-Royal Institute of Technology, Stockholm, Sweden; 12 Department of Neuroscience, Karolinska Institutet, Stockholm, Sweden; 13 The Department of Ophthalmology, Boston Children’s Hospital, Harvard Medical School, Boston, MA USA

## Abstract

**Background:**

Two risk factors for severe retinopathy of prematurity (ROP) in extremely preterm infants are thrombocytopenia and low levels of arachidonic acid (AA) and docosahexaenoic acid (DHA). To date, these risk factors have not been linked.

**Method:**

Infants born < 28 weeks gestational age (GA) from 2016 to 2019 were randomized to postnatal enteral AA/DHA supplementation or standard care (controls). Levels of AA and DHA, platelet counts ( < 100 × 10^9^/L defined as thrombocytopenia) and platelet-related proteins in the infants’ first four weeks of life were evaluated for their association with severe ROP.

**Results:**

The mean birthweight of 178 included infants was 806 ± 200 grams, and the mean GA was 25.6 ± 1.4 weeks. During the first four postnatal weeks, 20.2% of AA/DHA-supplemented infants had thrombocytopenia versus 27.7% of controls (*p* = 0.29). In infants with thrombocytopenia, fewer AA/DHA-supplemented infants developed severe ROP than non-supplemented controls, 29.4% (5/17) versus 65.4% (17/26) (*p* = 0.031). Thrombocytopenia and serum levels of AA and DHA correlated with several platelet-related proteins involved in angiogenesis and ROP, such as platelet-derived growth factor subunits A and B and vascular endothelial growth factor.

**Conclusions:**

AA and DHA supplementation is associated with less severe ROP in thrombocytopenic infants, possibly by modulating platelet activation and function.

**Impact:**

- Postnatal enteral supplementation with arachidonic acid (AA) and docosahexaenoic acid (DHA) to extremely preterm infants reduces the risk of severe retinopathy of prematurity (ROP) in infants with thrombocytopenia. The impact of AA and DHA might be, at least in part, mediated through altered platelet activation.
- We found that AA and DHA may reduce the risk of severe ROP, possibly by modulating platelet-related proteins involved in angiogenesis.
- Our findings strongly support that supplementing AA and DHA to extremely preterm infants is crucial and can significantly impact their health.

## Introduction

Retinopathy of prematurity (ROP) is a sight-threatening disease of the developing retina in infants born preterm.^1^ In the first phase of ROP, vasoactive growth factors, such as vascular endothelial growth factor A (VEGF-A) and platelets-derived growth factors (PDGFs), are suppressed in the hyperoxic retina and normal retinal vascularization is arrested. In the second phase of ROP, ischemia in the now avascular peripheral retina stimulates the production of these vasoactive growth factors. This prompts abnormal retinal neovascularisation, which can lead to retinal detachment and blindness.^2,3^

Thrombocytopenia has been identified as an independent risk factor for ROP.^4–7^ It has been suggested that platelets can locally stimulate or inhibit angiogenesis as they store and release angiogenesis regulators, such as VEGF-A and PDGFs.^8–10^ Thrombocytopenia is commonly found in preterm infants.^11^ The primary mechanism of postnatal thrombocytopenia is thought to be impaired megakaryocytopoiesis and platelet production,^12^ in addition to increased platelet consumption due to sepsis, necrotising enterocolitis (NEC), perinatal asphyxia and other infections.^11,13^ Preterm infant platelets are less reactive than platelets in term infants and adults, with markedly prolonged coagulation times and decreased granule release.^14–17^

Extremely preterm birth interrupts the last trimester *in utero* transfer to the fetus of long-chain polyunsaturated fatty acids (LC-PUFA), such as arachidonic acid (AA, 20:4 ω-6) and docosahexaenoic acid (DHA, 22:6 ω-3). AA and DHA are essential components of cell membranes in many cells and particularly important in neural cells, i.e. brain and retina.^18,19^ AA and DHA are also crucial in maintaining platelet structure, survival, apoptosis, and stem cell proliferation regulation.^20^ AA is the most abundant fatty acid in platelet membranes and important for platelet α-granule release.^21^ Dysregulation of lipid metabolism leads to substantial defects in platelet function,^21–25^ and long-term parenteral nutrition has been associated with LC-PUFA deficiency, platelet dysfunction, thrombocytopenia and ROP in infants.^26,27^ In a randomised study, we found that postnatal enteral supplementation with AA and DHA to extremely preterm infants reduced severe ROP by 50%.^28^ The specific relationship between LC-PUFAs and platelets and how they interact in the development of ROP is not known.^20,21^

This study investigated the association between levels of AA and DHA, thrombocytopenia, platelet-related proteins, and severe ROP in a cohort of extremely preterm infants in Sweden who received either enteral AA and DHA supplementation or standard care. We hypothesized that the beneficial effects of supplementation would include platelet counts and function, as LC-PUFAs are suggested to be involved in the functional maturation of platelets.

## Methods

This cohort study was a substudy of the Mega Donna Mega study, an open-label, randomized clinical trial (ClinicalTrials.gov Identifier: NCT03201588) conducted at three neonatal intensive care units in Sweden, including infants born at less than 28 weeks of gestational age (GA) in 2016 – 2019. The primary aim of the Mega Donna Mega study was to evaluate if an enteral fatty acid supplementation with AA and DHA could reduce severe ROP.

All included infants received nutritional support according to national guidelines and local policy (Supplementary Table 1). Details of the nutritional strategy has been published.^28,29^ The AA/DHA intervention aimed to correspond to fetal accretion of these lipids.^30^ Half of the infants were randomized to standard care (control, no placebo) and half to receive additional treatment with the trial oral supplement consisting of a triglyceride oil (Formulaid, DSM Nutritional Products Inc.) extracted from a filamentous fungus (*Mortierella alpina*) and marine microalgae (*Crypthecodinium cohnii*). The AA/DHA supplementation was initiated within 72 h after birth and given daily to 40 weeks post menstrual age (PMA). The supplement was given daily at a dose of 0.39 ml/kg (adjusted weekly according to infant weight), corresponding to 100 mg/kg/day of AA and 50 mg/kg/day of DHA. The LC-PUFA supplement was provided as a bolus through the nasogastric tube together with human milk (mother’s own milk or donor milk), preterm formula, or in the buccal cavity in connection to feeding. Details about this randomized clinical trial have been previously published,^28,29^ and additional information regarding the supplementation is found in the trial protocol (trial protocol in Supplement document 1). The trial protocol was approved by the Regional Ethics Review Board in Gothenburg (303-11 and T570-15) and the Swedish Ethical Review Authority (2020-02381). The principles of the Declaration of Helsinki were followed. This study followed the Consolidation Standards of Reporting Trials (CONSORT) reporting guidelines (Supplement document 2).

### Birth characteristics and neonatal infant morbidities

We recorded the birth characteristics of all infants, including sex, GA, and birth weight (BW). Neonatal morbidities, like bronchopulmonary dysplasia (BPD), intraventricular haemorrhage (IVH), sepsis and NEC, up to 40 weeks of PMA were recorded. BPD was defined as a need for supplemental oxygen at PMA 36 weeks. IVH was diagnosed by ultrasound on days 3 and 7 and graded according to Papile et al. and IVH grades 3–4 were defined as severe.^31^ Sepsis was defined as clinical symptoms and bacterial/fungal (non-viral) infection confirmed by positive blood culture unless the blood culture indicated Coagulase-negative staphylococci, diphtheroid, or a mixed bacterial flora, in which case an elevated C-reactive protein (CRP) > 20 mg/L was required. NEC diagnoses were based on clinical and radiological findings (Bell’s stage 2–3). To account for platelet-consuming conditions such as sepsis, NEC, other infections, asphyxia and inflammation, we considered an elevated CRP > 20 mg/L to be pathological and to reflect these conditions. The number of platelet transfusions was retrieved from the infants’ medical records.

ROP screening examinations followed a routine protocol consisting of dilated ocular fundus examinations. The examining ophthalmologists were masked for randomized supplementation. ROP was classified using the International Classification of Retinopathy of Prematurity.^32^ Treatment was provided according to the recommendations of the Early Treatment for Retinopathy of Prematurity Cooperative Group.^33^ ROP ≥ stage 3 and treated was defined as severe ROP.

### Blood sampling

According to local guidelines and clinical indications, routine blood samples for platelet count and CRP levels were drawn. All available platelet counts and CRP values were retrieved until 40 weeks PMA from the infants’ medical records. When multiple platelet counts and CRP values were available for an infant on the same day, the lowest platelet value and the highest CRP value during that day was used. We defined thrombocytopenia as a platelet count <100 × 10^9^/L. CRP levels > 20 mg/L were considered pathological, irrespective of the underlying condition. We registered thrombocytopenia and CRP > 20 mg/L in the first postnatal week, during one to four postnatal weeks, before 30 weeks PMA, and in and after 30 weeks PMA.

Levels of phospholipid fatty acid and platelet-related protein were retrospectively retrieved from the original study, where whole-blood samples (0.6 mL) for serum analysis were collected on postnatal days 0, 3, 7, 14, and 28 according to study protocol (Supplement document 1). Molar percentages of serum phospholipid fatty acids were determined by gas chromatography-mass spectrometry, as previously described.^34^ Platelet-related serum proteins were analysed using a multiplex PEA technology (Olink Bioscience, Uppsala, Sweden). The analysis covered 538 unique protein targets after removing duplicates and targets not passing quality controls. Six Olink Target 96-proteins panels were used (Cardiometabolic v.3603, Cardiovascular II v.5006, Cardiovascular III v.6113, Development v.3512, Metabolism v.3402, and Inflammation v.3022). Data acquisition and postprocessing procedures have been published.^35^ We identified proteins that, according to https://geneontology.org/, had the word “platelet” in the description of their biological process, cellular component or molecular function. We then identified 61 proteins as platelet-related (Supplementary Table 2).

### Statistical analysis

All analyses were performed in SAS version 9.4 (SAS Institute Inc., Cary, NC) and R version 4.3.1.

The analyses performed were exploratory, and a significance level 0.05 was applied. Mean, standard deviation (SD), median and range were presented for continuous variables and counts and percentages for categorical variables. For tests between two groups, Fisher’s exact test was used for dichotomous variables and Fisher’s non-parametric permutation test for continuous variables. Unadjusted logistic regression was applied when studying the risk for severe ROP by thrombocytopenia and CRP > 20 mg/L. Adjustments were made for GA, center and AA/DHA supplementation. Odds ratios (OR) with associated 95% confidence intervals (CI) were presented. For robustness, analyses were repeated for 1000 bootstrapped studies with replacement and the distribution of OR and *p*-values was presented.

In the analyses evaluating associations between mean platelet-related proteins and platelet counts, thrombocytopenia within 28 days, mean AA and mean DHA, all longitudinal variables were handled as a mean value of day 0 (actual day 0 or 1), day 7 (closest value between 2 and 10 days), day 14 (closest value between 11 and 20 days), and day 28 (closest value between 21 and 40 days). At least three out of four values were required. Linear regression models were applied specifying the platelet-related protein as an outcome variable, thrombocytopenia and AA and DHA levels as main effect variables in the separate analyses, and GA, center and randomized supplementation group as covariates if applicable. The outcome variables were standardized for SD to numerically compare the standardized beta estimates between different proteins. Adjustment for multiple testing was performed using the Benjamini-Hochberg approach for false discovery rate. The 20 platelet-related proteins which presented the most significant relationship to thrombocytopenia and AA or DHA levels after adjustment for multiple comparisons were shown in a forest plot.

## Results

### Clinical characteristics and postnatal morbidities

**Fig. 1:**
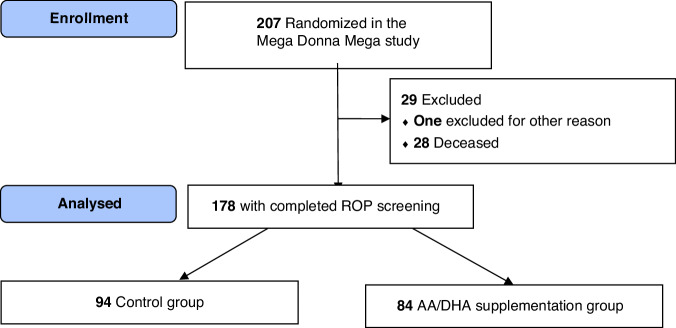
Flowchart of the study population. AA arachidonic acid, DHA docosahexaenoic acid, ROP retinopathy of prematurity.

In the original Mega Donna Mega study, 178 infants had completed ROP screening and were eligible for this follow-up study (Fig. 1). The infant birth characteristics and postnatal morbidities are described in Table 1. The infants had a mean GA at the birth of 25.6 ( ± 1.4) weeks and a mean BW of 806 ( ± 200) grams. In total, 51 infants (28.7%) developed severe ROP, AA/DHA supplemented infants less frequently than controls, 19.0% (16/84) versus 37.2% (35/94), *p* = 0.0081, as previously reported.^28^

**Table 1:** Infants’ characteristics, comorbidities, presence of thrombocytopenia, platelet transfusion, a CRP > 20 mg/L and sepsis during the study.

Variable	Total *N* = 178	Standard care *N* = 94	AA/ DHA supplement *N* = 84	*p*-value
**Infants characteristics**				
Gestational age (weeks)	25.6 ± 1.4 25.7 (22.6–27.9)	25.6 ± 1.4 25.8 (22.9–27.9)	25.6 ± 1.5 25.6 (22.6–27.9)	0.77
Birth weight (grams)	806 ± 200 786 (425–1345)	795 ± 195 775 (425–1330)	818 ± 205 796 (455–1345)	0.45
BPD	96 (53.9%)	48 (51.1%)	48 (57.1%)	0.45
IVH grade ≥ 3	21 (11.8%)	14 (14.9%)	7 (8.3%)	0.24
NEC	15 (8.4%)	10 (10.6%)	5 (6.0%)	0.29
Severe ROP (stage 3 ROP and treated)	51 (28.7%)	35 (37.2%)	16 (19.0%)	**0.0081**
**Thrombocytopenia present**				
< 1 week PNA	30 (16.9%)	21 (22.3%)	9 (10.7%)	**0.046**
< 4 weeks PNA	43 (24.2%)	26 (27.7%)	17 (20.2%)	0.29
< 30 weeks PMA	46 (25.8%)	28 (29.8%)	18 (21.4%)	0.23
≥ 30 weeks PMA	18 (10.1%)	8 (8.5%)	10 (11.9%)	0.47
**Platelet transfusion present**				
< 1 week PNA	7 (3.9%)	3 (3.2%)	4 (4.8%)	0.71
< 4 weeks PNA	14 (7.9%)	7 (7.4%)	7 (8.3%)	1.00
< 30 weeks PMA	17 (9.6%)	7 (7.4%)	10 (11.9%)	0.44
≥ 30 weeks PMA	7 (4.0%)	3 (3.2%)	4 (4.8%)	0.71
**Number of platelet transfusion**				
<1 week PNA	1.4 ± 0.8 (*n* = 7)	1.7 ± 1.2 (*n* = 3)	1.3 ± 0.5 (*n* = 4)	0.71
<4 weeks PNA	3.1 ± 3.2 (*n* = 14)	4.4 ± 4.1 (*n* = 7)	1.7 ± 1.3 (*n* = 7)	0.15
<30 weeks PMA	2.8 ± 3.0 (*n* = 17)	4.4 ± 4.1 (*n* = 7)	1.6 ± 1.1 (*n* = 10)	**0.039**
≥30 weeks PMA	4.4 ± 4.8 (*n* = 7)	2.3 ± 2.3 (*n* = 3)	6.0 ± 5.9 (*n* = 4)	0.46
**CRP** > **20** **mg/L event present**				
< 1 week PNA	21 (11.8%)	14 (14.9%)	7 (8.3%)	0.24
< 4 weeks PNA	50 (28.1%)	31 (33.0%)	19 (22.6%)	0.14
< 30 weeks PMA	55 (30.9%)	30 (31.9%)	25 (29.8%)	0.87
≥ 30 weeks PMA	43 (24.2%)	24 (25.5%)	19 (22.6%)	0.73
**Sepsis event present**				
< 1 week PNA	5 (2.8%)	3 (3.2%)	2 (2.4%)	1.00
< 4 weeks PNA	30 (16.9%)	19 (20.2%)	11 (13.1%)	0.23
< 30 weeks PMA	35 (19.7%)	20 (21.3%)	15 (17.9%)	0.58
≥ 30 weeks PMA	14 (7.9%)	12 (12.8%)	2 (2.4%)	**0.011**

Statistically significant *p*-values are in bold.

Thrombocytopenia is defined as a platelet count < 100 × 10^9^/L.

Data are presented as mean ± standard deviation, median (range) number of observations, or number (percentage).

Fisher’s exact test was used for the test between two groups concerning dichotomous variables, and for continuous variables, Fisher’s non-parametric permutation test.

*BPD* bronchopulmonary dysplasia, *CRP* C-reactive protein, *IV*H intraventricular haemorrhage, *NEC* necrotizing enterocolitis, *ROP* retinopathy of prematurity, *PMA* postmenstrual age, *PNA* postnatal age.

### Longitudinal serum levels of long-chain polyunsaturated fatty acids

Longitudinal serum levels of DHA and AA have previously been described in detail.^28^

### Platelet counts and platelet transfusions

**Fig. 2:**
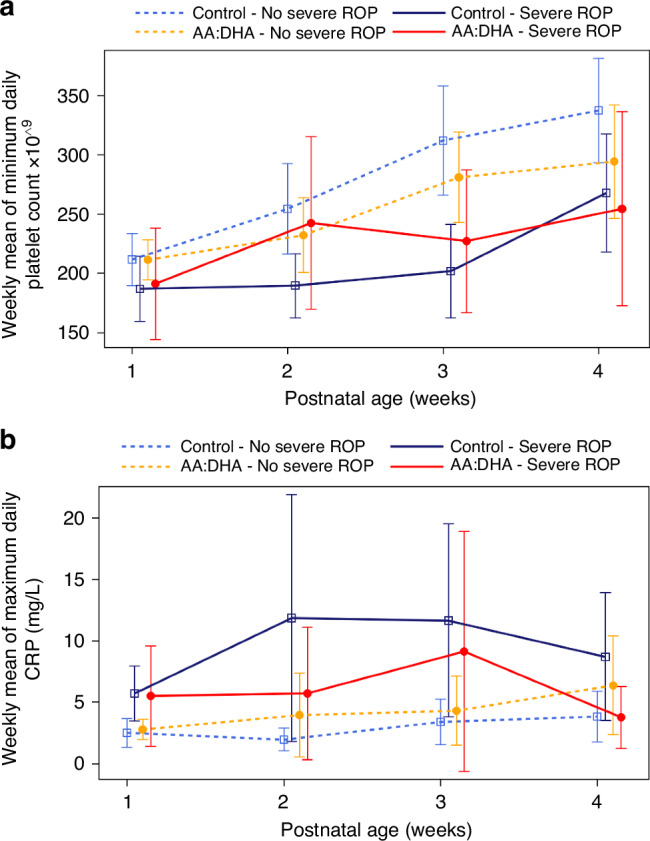
Platelet counts and CRP levels first four weeks of life. Platelet counts x 10^9^ and CRP (mg/L) over postnatal weeks by severe ROP and AA/DHA supplementation group, (**a**) Weekly mean of minimum daily platelet counts with 95% CI, (**b**) weekly mean of maximum daily CRP with 95% CI. AA arachidonic acid, DHA docosahexaenoic acid, CRP C-reactive protein, ROP retinopathy of prematurity, TCP thrombocytopenia.

The weekly mean of minimum daily platelet count in infants stratified by AA/DHA supplementation and the presence of severe ROP over time is presented in Fig. 2a (first four postnatal weeks) and Supplementary Fig. 1A (PMA weeks). During the first week of life, AA/DHA supplemented infants had significantly less thrombocytopenia than controls, 10.7% (9/84) versus 22.3% (21/94) (*p* = 0.046). However, there was no significant difference in the presence of thrombocytopenia over the first four postnatal weeks between AA/DHA supplemented infants and controls, 20.2% (17/84) versus 27.7% (26/94) (*p* = 0.29). The number of infants requiring platelet transfusion in the two groups was comparable; however, before 30 weeks of PMA, control infants received significantly more transfusions per infant than supplemented (4.4 ± 4.1versus 1.6 ± 1.1, *p* = 0.039), Table 1.

### CRP

The weekly mean of maximum daily CRP in preterm infants stratified by AA/DHA supplementation and the presence of severe ROP over time is presented in Fig. 2b (first four postnatal weeks) and Supplementary Fig. 1B (PMA weeks). There were no significant differences in the presence of CRP > 20 mg/L between AA/DHA-supplemented infants and controls during the study period.

### Thrombocytopenia and high CRP in relation to severe ROP

**Fig. 3:**
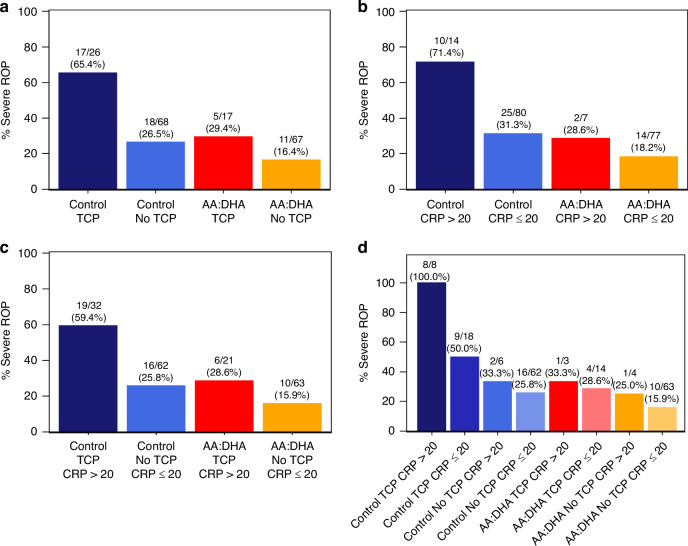
Thrombocytopenia and/or high CRP and severe ROP. Frequency of severe ROP according to AA/DHA supplementation or standard care stratified by (**a**) presence of thrombocytopenia during the first four postnatal weeks, (**b**) levels of CRP ≤ or > 20 mg/L during the first postnatal week, (**c**) presence of thrombocytopenia during the first four postnatal weeks or levels of CRP ≤ or > 20 mg/L during the first postnatal week, (**d**) combinations of thrombocytopenia or not during the first four postnatal weeks and/or CRP ≤ or > 20 mg/L during the first postnatal week. AA arachidonic acid, DHA docosahexaenoic acid, CRP C-reactive protein, ROP retinopathy of prematurity, TCP thrombocytopenia.

When stratifying AA/DHA-supplemented infants and controls according to the presence of thrombocytopenia in the first four postnatal weeks, AA/DHA-supplemented infants with thrombocytopenia had less severe ROP than controls with thrombocytopenia, 29.4% (5/17) versus 65.4% (17/26), (*p* = 0.031), respectively. Figure 3a–d shows proportions of severe ROP in the AA/DHA supplemented and control infants according to high CRP (during the first postnatal week) or/and thrombocytopenia (during the first four postnatal weeks). Control infants with thrombocytopenia and with high CRP had the highest rate of severe ROP, 100% (8/8), while AA/DHA supplemented infants without thrombocytopenia and without high CRP had the lowest rate, 15.9% (10/63).

Table 2 presents unadjusted and adjusted logistic regression for the association between thrombocytopenia and a high CRP ( > 20 mg/L) event, respectively, with severe ROP during different periods. In unadjusted logistic regression, thrombocytopenia was found to be a significant risk factor for severe ROP throughout the infant’s whole neonatal course; however, the significance and impact were reduced in some periods when adjusting for GA, center, and AA/DHA supplementation. Early thrombocytopenia during the first four postnatal weeks was found to be a significant risk factor for severe ROP in both unadjusted (OR 3.83, 95% CI 1.85–7.91, *p* = 0.0003) and adjusted analyses (OR 2.35, 95% CI 1.00–5.48, *p* = 0.049) as did later thrombocytopenia ≥30 weeks PMA (OR 4.71, 95% CI 1.71–12.98, *p* = 0.0027) in unadjusted and in adjusted analysis (OR 3.68, 95% CI 1.07–12.62, *p* = 0.038).

**Table 2:** Unadjusted and adjusted logistic regression for the association between thrombocytopenia, a CRP > 20 mg/L, and sepsis with severe ROP.

			Unadjusted logistic regression		Adjusted logistic regression^a^	
Variable	Value	Number (%) of severe ROP	OR (95% CI)	*p*-value	OR (95% CI)	*p*-value
**Thrombocytopenia** < **100** **×** **10**^**9**^**/L**						
< 1 week PNA	No	36 (24.3%)				
	Yes	15 (50.0%)	3.11 (1.39–6.98)	**0.0059**	1.54 (0.59–3.97)	0.38
< 4 weeks PNA	No	29 (21.5%)				
	Yes	22 (51.2%)	3.83 (1.85–7.91)	**0.0003**	2.35 (1.00–5.48)	**0.049**
< 30 weeks PMA	No	27 (20.5%)				
	Yes	24 (52.2%)	4.24 (2.07–8.69)	**<0.0001**	2.26 (0.98–5.22)	0.06
≥ 30 weeks PMA	No	40 (25.0%)				
	Yes	11 (61.1%)	4.71 (1.71–12.98)	**0.0027**	3.68 (1.07–12.62)	**0.038**
**CRP** > **20** **mg/L**						
< 1 week PNA	No	39 (24.8%)				
	Yes	12 (57.1%)	4.03 (1.58–10.30)	**0.0035**	4.24 (1.32–13.57)	**0.015**
< 4 weeks PNA	No	27 (21.1%)				
	Yes	24 (48.0%)	3.45 (1.72–6.94)	**0.0005**	2.43 (1.07–5.52)	**0.033**
< 30 weeks PMA	No	24 (19.5%)				
	Yes	27 (49.1%)	3.98(1.99–7.94)	**<0.0001**	2.65 (1.18–5.95)	**0.018**
≥ 30 weeks PMA	No	35 (25.9%)				
	Yes	16 (37.2%)	1.69 (0.82–3.51)	0.16	1.24 (0.53–2.93)	0.62
**Sepsis event**						
< 1 week PNA	No	48 (27.7%)				
	Yes	3 (60.0%)	3.91 (0.63–24.11)	0.14	3.33 (0.40–27.34)	0.26
< 4 weeks PNA	No	39 (26.4%)				
	Yes	12 (40.0%)	1.86 (0.82–4.22)	0.14	1.64 (0.59–4.54)	0.34
<30 weeks PMA	No	34 (23.8%)				
	Yes	17 (48.6%)	3.03 (1.41–6.52)	**0.0046**	2.48 (0.96–6.41)	0.06
≥ 30 weeks PMA	No	45 (27.4%)				
	Yes	6 (42.9%)	1.98 (0.65–6.04)	0.23	2.49 (0.66–9.42)	0.18

Statistically significant *p*-values are in bold.

^a^Models are adjusted for GA, center, and AA/DHA supplement.

*AA* arachidonic acid, *CI* confidence interval, *CRP* C-reactive protein, *DHA* docosahexaenoic acid, *GA* gestational age, *OR* odds ratio, *PMA* postmenstrual age, *PNA* postnatal age, *ROP* retinopathy of prematurity.

The presence of CRP > 20 mg/L as a risk factor for severe ROP was significant during the first postnatal week, the first four postnatal weeks and before 30 weeks of PMA in unadjusted as well as adjusted logistic regression, with the most significant impact if present during the first postnatal week (OR 4.24, 95% CI 1.32–13.57, *p* = 0.015, adjusted analyses).

By bootstrapping 1000 studies with replacement, these analyses were shown to hold as presented following the robustness analyses in the Supplementary Table 3.

### Thrombocytopenia and high CRP and levels of AA and DHA

**Fig. 4:**
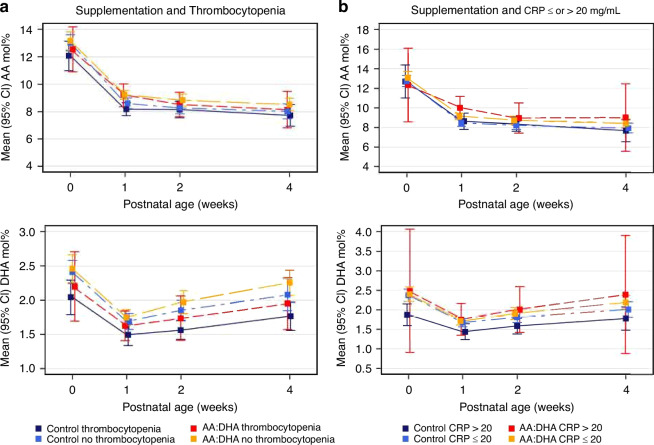
AA and DHA levels during first four weeks of life. Mean serum AA and DHA with 95% CI per supplementation group and (**a**) thrombocytopenia during the first four postnatal weeks, (**b**) CRP ≤ or > 20 mg/L during the first postnatal week. AA arachidonic acid, DHA docosahexaenoic acid, CRP C-reactive protein, ROP retinopathy of prematurity.

Figure 4 shows the mean molar percent (mol%) of AA and DHA by supplementation group, the presence of thrombocytopenia during the first four postnatal weeks (left panel) and the presence of any measurement of CRP > 20 mg/L during the first week of life or not (right panel). The highest levels of DHA were notable in the AA/DHA supplemented infants without thrombocytopenia and the lowest in the control infants with thrombocytopenia.

### Platelet-related proteins and their association with thrombocytopenia and levels of AA and DHA

Supplementary Table 4 presents the associations between platelet-related proteins, thrombocytopenia and levels of AA and DHA after adjustments for GA, center, and supplementation group (unless main effect variable).

**Fig. 5:**
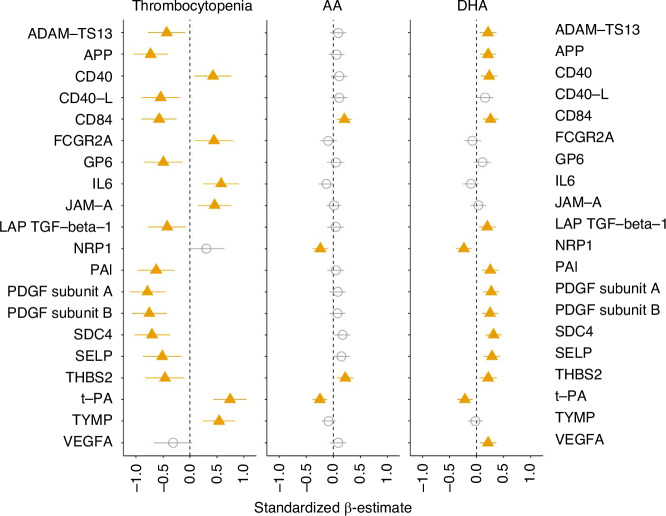
Top 20 platelet related proteins. Presentation of 20 platelets-related proteins with the most prominent associations to platelet counts, presence of thrombocytopenia within the first four postnatal weeks and levels of AA and DHA. Significant associations (*p* > 0.05) are shown in filled yellow triangles, and non-significant associations are shown in open circles. Error bars show 95% CI. AA arachidonic acid, DHA docosahexaenoic acid.

Figure 5 shows the top 20 platelet-related proteins with the most prominent association with thrombocytopenia and levels of AA and DHA. In Supplementary Fig. 2, we present examples of longitudinal protein levels per AA/DHA supplementation group and thrombocytopenia during the first four postnatal weeks.

Briefly, thrombocytopenia showed a significant negative association with PDGF-A and B, Amyloid-beta precursor protein (APP), SLAM family member 5 (CD84), Transforming growth factor beta-1 (LAP-TGF-beta-1), Plasminogen activator inhibitor 1 (PAI), Syndecan-4 (SDC4) and Thrombospondin-2 (THBS2), all proteins known to be involved in ROP development.^36–40^

Both AA and DHA levels showed a positive association with the previously mentioned THBS2 and CD84 and a negative association with t-PA^41,42^ and Neuropilin-1 (NRP1)^43,44^ known to be involved in angiogenesis. DHA showed additional positive association with other proteins involved in angiogenesis, like VEGF-A and PDGF-A. Furthermore, P-Selectin (SELP), a protein specific for α-granules and platelet degranulation, showed a negative association with thrombocytopenia and a positive to levels of DHA.^45–47^

AA/DHA supplementation did not show any associations with platelet-related protein following Benjamini-Hochberg FDR adjustment for multiple testing and model adjustment for GA, center, and supplementation group.

## Discussion

This study confirms thrombocytopenia as a risk factor for severe ROP in extremely preterm infants. Postnatal enteral supplementation with AA/DHA did not significantly affect the proportion of thrombocytopenic infants. However, AA/DHA supplementation was associated with a reduced rate of severe ROP in infants with thrombocytopenia compared to controls receiving standard care. These findings suggest a LC-PUFA-induced change in platelet activity and function. Levels of AA, DHA, and early thrombocytopenia were associated with specific platelet-related proteins known to be involved in angiogenesis and ROP development. We hypothesize that AA/DHA supplementation modulates the impact of thrombocytopenia as a risk factor for severe ROP secondary to changes in platelet function.

### The role of platelets in ROP pathology

We and others have previously reported thrombocytopenia in the first and second phases of the ROP disease in infants requiring ROP treatment.^4–7^ This study focused on the first four weeks of preterm life, corresponding to the first phase of ROP with vessel growth suppression when pro-angiogenetic proteins are downregulated.

Platelets were initially recognized primarily for their role in thrombosis and hemostasis. More recently, proteomic studies have revealed that platelets release α-granules that carry hundreds of bioactive proteins involved in inflammation, atherosclerosis, immune response, wound healing, and angiogenesis.^10^ Platelets contain both pro- and anti-angiogenic proteins in their α-granules, regulating angiogenesis when released.^8,10,48–50^ Formation and development of the retinal vasculature, including sprouting angiogenesis, vascular remodelling and vessel maturation, is a complex process interrupted by preterm birth.^51^ The most established pro-angiogenetic proteins involved in ROP and associated with platelets are PDGF-A and B and VEGF-A.^9,36,52^ In our study, we found that several additional platelet-related angiogenetic proteins known to be involved in ROP also were correlated with levels of LC-PUFAs such as APP, CD84, LAP-TGF-beta-1, PAI and THBS2,^36–39^ suggesting that sufficient levels of platelets and LC-PUFAs are essential in preventing the first phase of ROP, and in promoting continued physiological vessel growth in the retina.

Both DHA and AA levels were negatively associated with NRP1 and t-PA levels. These results are interesting as NRP1 and t-PA promote angiogenesis.^41,42,44^ Many angiogenetic proteins exhibit pro- and anti-angiogenic effects depending on ligands and co-protein expression. In an animal model, exogenous t-PA markedly suppressed corneal neovascularization.^53^

### Importance of AA and DHA in platelet functions

The connection we found between LC-PUFA and thrombocytopenia is not unexpected; both in vitro and in vivo studies have demonstrated that AA and DHA can increase megakaryocyte migration, promote platelet formation, and modify platelet functions by integrating into the cell membranes.^20–23,54^ In adults, supplementation with either AA or DHA results in increases in the corresponding fatty acid in platelets, possibly due to their incorporation into bone marrow megakaryocytes.^55,56^ Studies investigating relationships between LC-PUFAs and platelets in infants or preterm infants are scarce. In the 1970s, Freidman et al. and Calman et al. reported that essential fatty acid deficiency in neonates is characterized by poor growth, dermatitis, and thrombocytopenia.^26,27^ We previously published that AA and DHA levels and platelet counts are associated in preterm infants with ROP.^57^ AA/DHA-supplemented infants in this study have a slightly lower occurrence of thrombocytopenia than controls, but not significantly so. When evaluating levels of AA and DHA, the control infants with thrombocytopenia had the lowest levels of AA and DHA, and the supplemented infants without thrombocytopenia had the highest levels.

AA is the most common fatty acid in platelet membranes and is a crucial mediator of platelet activity.^24,58^ AA influences the release of PDGF, and treatment with AA increases VEGF levels in a mouse model.^59,60^ In our study, SELP was positively associated with DHA levels during the first four postnatal weeks, suggesting enhanced activation and degranulation.^45^ There are scarce studies about the role of LC-PUFAs in preterm infant platelets^20^; however, our study demonstrates that AA and DHA have a fundamental role in platelet function and possibly the secretion of granules. Preterm infant platelets are less reactive, with markedly prolonged coagulation times and decreased granule secretion, than term infant and adult platelets.^14–17^ There is an age-dependent change in preterm infant platelet reactivity after birth. This might further complicate the interpretation and application of studies on platelet activity after fatty acid supplementation in adults.^16^

### AA/DHA supplementation and ROP

When an infant is born preterm, neither human milk nor formula recreates the in-utero transfer or meets the recommended intake of ω-6 and ω-3 LC-PUFAs, including AA and DHA.^61^ Adequate levels of AA and DHA are crucial for normal retinal and brain development in preterm infants, and deficiencies have been associated with long-term visual and cognitive sequelae.^62–65^ Studies concerning the benefit of parenteral and enteral supplementation of ω-3 LC-PUFAs in preterm infants on ROP development have been inconsistent, reflecting the complexity of supplementation.^34,66–70^ Only a few studies have explored combined supplementation with both AA and DHA in extremely preterm infants. Moltu et al. used a similar supplementation strategy with AA and DHA as in the Mega Donna Mega trial and reported that severe ROP was reduced by half in the intervention group, although the number of included infants was too low to show statistical significance.^71^ Henriksen et al. provided AA and DHA to infants with BW < 1500 g but found no effect of supplementation on treated ROP, however, the fatty acids were given at a relatively low dose.^72^ We have previously reported a 50% reduction in severe ROP in AA/DHA enteral supplemented infants in the current cohort, Mega Donna Mega trial. We have retrospectively reported higher mean daily serum levels of DHA during the first four weeks of life were associated with less severe ROP but only in infants with sufficiently high AA levels, supporting that both AA and DHA may contribute to protection against ROP.^28,73,74^ Both ω-6 and ω-3 LC-PUFA were crucial in protecting against retinal neurovascular dysfunction in phase I ROP in an experimental mouse model.^75^ We hope an ongoing clinical trial (ClinicalTrials NCT05380401) with combined AA and DHA supplementation will shed further light on the role of these LC-PUFAs in ROP development.

### Increased CRP as a risk factor for severe ROP

In this retrospective study, we primarily focused on thrombocytopenia as a risk factor for severe ROP. However, we also evaluated episodes of high CRP, which we consider to reflect platelet-consuming morbidities and established risk factors for severe ROP such as NEC, sepsis, other infections and inflammatory conditions. High CRP levels were found to be a significant independent risk factor for severe ROP and add to thrombocytopenia risk, especially in control infants. The relationship between thrombocytopenia and infection/inflammation and the risk for severe ROP has previously been described.^6^

### Strengths

The main strength of this randomized controlled study was its multicenter and longitudinal design.

### Limitations

This explorative follow-up study lacked an a priori statistical analysis plan. A limitation is that platelet counts and CRP were sampled according to clinical indication and thus not at specified time points as the LC-PUFAs and platelet-related proteins. In addition, as the vulnerable infants at risk of thrombocytopenia were more likely sampled, this may constitute a bias.

## Conclusion

In summary, our study confirms that thrombocytopenia is a risk factor for severe ROP in extremely preterm infants. Supplementation with AA/DHA did not affect the rate of thrombocytopenia during the neonatal period. However, the impact of early thrombocytopenia as a risk factor for severe ROP was reduced in AA/DHA-supplemented infants. Levels of AA, DHA, and early thrombocytopenia were associated with specific platelet-related proteins known to be involved in angiogenesis. Our findings suggest that LC-PUFA supplementation may reduce severe ROP rates by modulating platelet-related proteins involved in angiogenesis.

## Supplementary information

CONSORT-2010-Checklist

Corrected supplement 1

Supplementary figure 1

Supplementary figure 2

Supplementary Table 1

Supplemental Table 2

Supplemental Table 3

Supplemental Table 4

## Data Availability

The data that support the findings of this study are available from the corresponding author, upon reasonable request.
