# Supplementary figures and images for: Platelet characteristics in extremely preterm infants after fatty acid supplementation: a randomized controlled trial
Source: Pediatr Res. 2024 Dec 19;98(2):680–9. doi: 10.1038/s41390-024-03775-3 (PMC12454127; doi:10.1038/s41390-024-03775-3)

A)

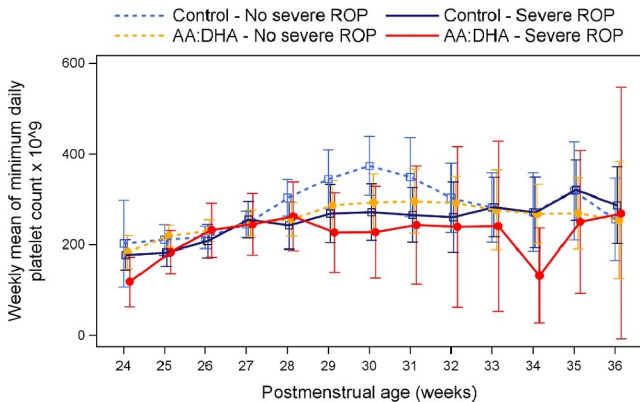

B)

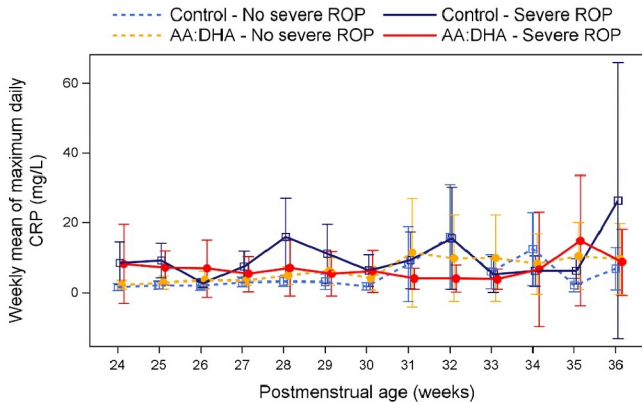

Supplement: Supplementary file 3 — Supplementary figure 1 [file 41390_2024_3775_MOESM3_ESM.pdf]
