# Supplementary material for: Platelet characteristics in extremely preterm infants after fatty acid supplementation: a randomized controlled trial
Source: Pediatr Res. 2024 Dec 19;98(2):680–9. doi: 10.1038/s41390-024-03775-3 (PMC12454127; doi:10.1038/s41390-024-03775-3)
Supplement: Supplementary file 4 — Supplementary figure 2 [file 41390_2024_3775_MOESM4_ESM.pdf]

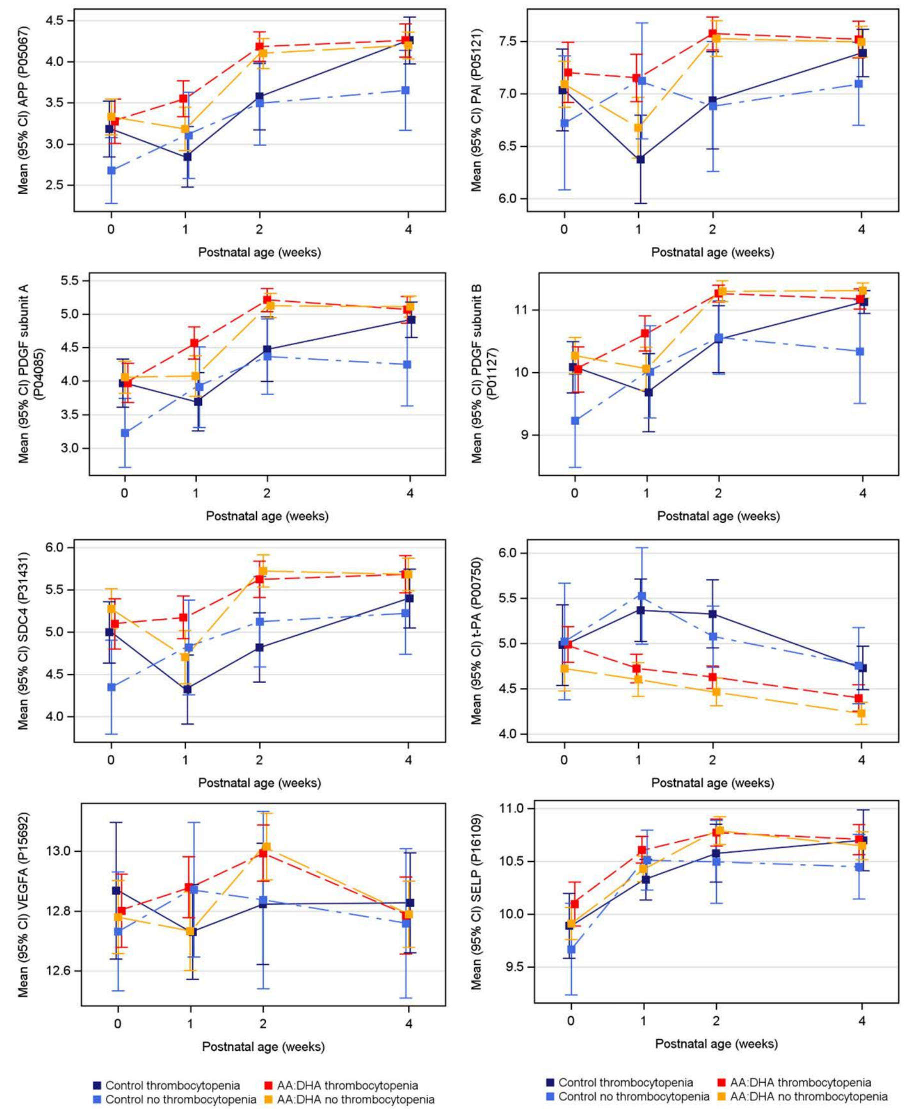

Mean (95% CI) APP (P05067)
Mean (95% CI) PAI (P05121)
Mean (95% CI) PDGF subunit A (P04085)
Mean (95% CI) PDGF subunit B (P01127)
Mean (95% CI) SDC4 (P31431)
Mean (95% CI) t-PA (P00750)
Mean (95% CI) VEGFA (P15692)
Mean (95% CI) SELP (P16109)
Postnatal age (weeks)
0
1
2
4
Control thrombocytopenia
Control no thrombocytopenia
AA:DHA thrombocytopenia
AA:DHA no thrombocytopenia
