# Supplementary material for: Platelet characteristics in extremely preterm infants after fatty acid supplementation: a randomized controlled trial
Source: Pediatr Res. 2024 Dec 19;98(2):680–9. doi: 10.1038/s41390-024-03775-3 (PMC12454127; doi:10.1038/s41390-024-03775-3)
Supplement: Supplementary file 5 — Supplementary Table 1 [file 41390_2024_3775_MOESM5_ESM.pdf]

**Supplemental Table 1: Nutritional support**				
**Variable**	**Total N=178**	**Standard care N=94**	**AA/ DHA supplement N=84**	***p*-value**
Energy intake (kcal/kg/day) days 2-28	124.3±14.1	123.3±13.5	125.3±14.9	0.34
Lipids intake (g/kg/day) days 2-28	6.0±1.3	5.9±1.3	6.1±1.3	0.32
Protein intake (g/kg/day) days 2-28	3.8±0.4	3.8±0.4	3.8±0.4	0.57
Carbohydrates intake (g/kg/day) days 2-28	13.1±1.2	13.1±1.0	13.2±1.5	0.62
**Time to reach full enteral feeds***				
Days	9.0 (13.0−18.0)	9.0 (13.0−22.0)	9.0 (12.0−16.0)	0.16
**Parenteral nutrition days 2-28****				
Days	8.0 (12.0−18.0)	8.0 (13.0−19.0)	7.5 (11.5−16.0)	0.15

* first day of life with enteral fluid intake ≥150 mL/kg/day.
** days with parenteral amino acids and/or lipids and enteral feeds ≤150mL/kg/day.
Data are presented as mean±standard deviation or median (25th – 75th percentile)
Student's T-test and Mann-Whitney U-test were used for the test between groups.
